# The emerging role of EGFL6 in angiogenesis and tumor progression

**DOI:** 10.7150/ijms.45129

**Published:** 2020-05-25

**Authors:** Jing Kang, Juanjuan Wang, Jihua Tian, Ruyi Shi, Hongyan Jia, Yanhong Wang

**Affiliations:** 1 Department of Microbiology and Immunology, Shanxi Medical University, Taiyuan, Shanxi, China.; 2 Department of Cell biology and Genetics, Shanxi Medical University, Taiyuan, Shanxi, China.; 3 Department of General Surgery, First Hospital of Shanxi Medical University, Taiyuan, Shanxi, China.

**Keywords:** EGFL, EGFL6, angiogenesis, tumorigenesis, biomarkers

## Abstract

Epidermal growth factor-like domain-containing protein 6 (EGFL6) belongs to the epidermal growth factor (EGF) superfamily. EGFL6 is expressed at higher levels in embryos and various malignant tumors than in normal tissues. Recent studies suggest that EGFL6 participates in the development of a variety of tumors. In this review, we summarize findings that support the role for EGFL6 in tumor proliferation, invasion and migration. Furthermore, our review results indicate the mechanism of EGFL6 activity angiogenesis. We also describe work toward the preparation of monoclonal antibodies against EGFL6. Altogether, the work of this review promotes understanding of the role of EGFL6 in tumor development, the mechanism of that action, and the potential of EGFL6 as a therapeutic target for tumor prevention and treatment.

## Introduction

Tumor growth, development, and metastasis depend on the formation of new blood vessels. As tumor size increases, new vasculature is required to provide blood flow and nutrients to the growing malignancy, a process termed angiogenesis. Tumor angiogenesis has become a hotspot of basic and clinical research. The most well-researched angiogenesis factor is vascular endothelial growth factor (VEGF). A variety of anti-angiogenic drugs have been developed against VEGF. Bevacizumab, a monoclonal antibody that blocks VEGF-A activity, is the most widely used anti-angiogenic drug. However, adverse reactions to Bevacizumab such as hypertension, hemorrhagic shock, and traumatic wound healing have become important issues. Further, in view of the effects of anti-VEGF drugs on wound healing, such drugs are not suitable for use during the perioperative period. Therefore, finding alternative targets for anti-tumor angiogenesis therapy has become critical.

Epidermal growth factor-like domain-containing protein 6 (EGFL6), a member of the epidermal growth factor (EGF) superfamily, plays a role in the regulation of cell cycle, proliferation, and development. EGFL6 is up-regulated in a variety of tumor tissues and promotes tumor angiogenesis. Further, EGFL6 expression levels are low or absent in normal tissues and blocks EGFL6 does not affect normal wound healing. Results of previous studies indicate that EGFL6 is closely related to tumor development. The mechanism of action of EGFL6 in tumor formation and development is a topic of active research. Findings from this research are expected to provide new biomarkers and therapeutic targets for tumor prevention and treatment.

### EGFL6 gene and protein structure

Epidermal growth factor-like (EGFL) domains contain 30-40 amino acid residues that have significant homology to EGF. Proteins with single or multiple EGFL domains can activate extracellular signal-regulated kinase (ERK), nuclear factor kappa B (NFĸB), mitogen-activated protein kinase (MAPK), protein kinase B (PKB), Notch and other important signal transduction pathways. The binding of EGFL proteins to their receptors triggers a wide range of biological functions, including proliferation, differentiation, apoptosis, adhesion, and migration 1. Further, activating different signal pathways, EGFL proteins participate in angiogenesis and the formation and development of various tumors. EGFL family members include EGFL2, EGFL3, EGFL5, EGFL6, EGFL7, EGFL8, and EGFL9 2. At present, much research has been done on EGFL7. This protein is considered to be a secretory factor derived from vascular endothelial cells. EGFL7 can play a role in regulating vascular endothelial cell adhesion and migration and in inhibiting vascular smooth muscle cell migration 3. EGFL7 is expressed in a variety of tumors, including tumors of malignant glioma 4 and tumors of cancers of the liver 5, breast 6, lung 7, and pancreas 8. EGFL6 has received extensive attention in recent years due to its roles in angiogenesis and tumor occurrence and development.

EGFL6, also known as MAEG, W80, was first described by Yeung and colleaguesin 1999 9. They used DNA molecular hybridization screening and identified EGFL6 as a novel EGF repeat superfamily member. The human EGFL6 gene is located on the Xp22.2 chromosome. It has 12 exons and 19 introns. This gene can produce two mRNA transcript variants. These variants use an alternate splice sites in the coding region. Variant 2 encodes isoform 2, which is 1 amino acid longer than isoform 1. The CDS region of splicing isoform 1 is 1662 bp in length and encodes 553 amino acids. The CDS region of splicing isoform 2 is 1665 bp in length and encodes 554 amino acids. The EGFL6 gene encodes a protein with a relative molecular mass of 63.01 KDa. The structure contains a signal peptide sequence. Chim and colleagues demonstrated by co-immunoprecipitation and Western blot analyses that EGFL6 is a secreted protein and exists as a homomeric complex 10.

**Figure 1:**
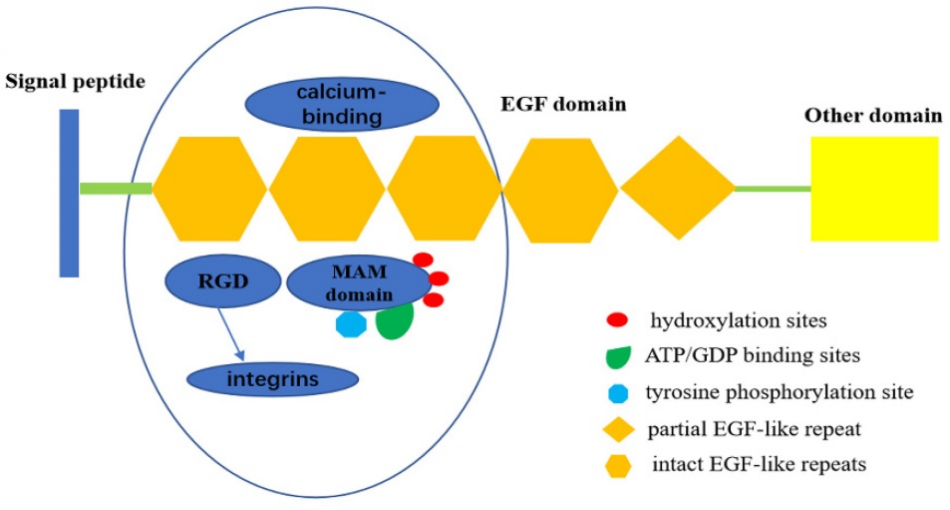
Structure of EGFL6.EGFL6 consists of four intact EGF-like repeats and one partial EGF-like repeat, Three of them have a calcium-binding consensus sequence, an arg-gly-asp integrin binding motif (RGD), and a MAM domain. RGD motif in EGFL6 can interact with integrins to activate some related signal pathways. MAM domain has three hydroxylation sites and ATP/GDP binding sites, as well as a potential tyrosine phosphorylation site,which indicates that MAM domain can be served as kinase substrate to phosphorylate it ,therefore, regulates the expression and function of EGFL6.

The EGF repeat region in EGFL6 consists of four intact EGF-like repeats and one partial EGF-like repeat. EGFL6 is highly expressed in certain tumor and fetal tissues, indicating its role as a growth factor. Three of the EGF-like domains of EGFL6 have a calcium-binding consensus sequence, an arg-gly-asp integrin binding motif (RGD), and a MAM domain. The presence of the RGD motif in EGFL6 predicts interaction with integrins 11. The MAM domain is associated with cell adhesion 12. There are also three hydroxylation sites, three ATP/GDP binding sites, and a potential tyrosine phosphorylation site in the MAM domain. The presence of these sites indicate that MAM domain can be used as a kinase substrate to phosphorylate and regulate the expression and function of EGFL6 13,14 (Figure 1).

According to the results of gene homology alignment on NCBI, the EGFL6 gene is conserved in chimpanzee, Rhesus monkey, dog, cow, mouse, rat, chicken, zebrafish, and frog. Moreover, studies have shown that EGFL6 is specifically expressed in some tumors but not in normal adult tissues. Therefore, EGFL6 gene products represent potential markers of malignant tumors 15 and are candidate targets for small molecule or antibody therapeutic agents for treating certain tumors.

## EGFL6 in vascular development

### EGFL6 involvement in embryonic angiogenesis

EGF-like proteins play a variety of roles in angiogenesis and endothelial cell functions. Previous studies have shown that EGFL7 is a secretory factor derived from vascular endothelial cells that plays a role in regulating vascular endothelial cell adhesion and migration and inhibits vascular smooth muscle cell migration 16. EGFL7 is important for regulating tubulogenesis in zebrafish and for controlling vascular patterning and integrity in mice 17-22. Its function in blood vessel development is mediated, at least in part, through modulation of Notch signaling and Akt/ERK activation 23. EGFL6 and EGFL7 belong to the EGFL superfamily and have similar structural and functional characteristics. Wang and colleagues, using whole-mount in situ hybridization and immunostaining have confirmed that the RGD domain in EGFL6 can promote angiogenesis in zebrafish 24. Integrin β1 is one of the receptors that recognize RGD motifs. EGFL6 regulates embryonic angiogenesis that depends on RGD domain through integrin β1, activating Akt/ERK signaling pathway. EGFL6 upregulates functional angiogenesis, indicating that EGFL6 participates in vertebrate embryonic vascular development 25. Further *in vivo* studies are needed for the functional analysis of EGFL6 involved in vertebrate embryonic vascular development.

### EGFL6 promotes endothelial cell migration and angiogenesis by activating ERK

Tumor growth and metastasis depend on tumor angiogenesis. Vascular endothelial cell migration is an important part of tumor angiogenesis. Chim and colleagues 10 confirmed that EGFL6 can induce endothelial cell migration and angiogenesis by conducting wound healing, transwell migration assay, tube formation assay and Chick Embryo Chorioallantoic Membrane Assay (CAM) *in vitro* and *in vivo*. CAM assay which is an *in vivo* angiogenesis assay allowing blood vessel formed on chorioallantoic membrane. these studies showed that EGFL6 is involved in various aspects of angiogenesis: promotion endothelial cell migration by a scratch wound healing assay and a transwell migration assay, enhancement of tube-like structure by a tube formation assay, formation of new blood vessels in a CAM assay. They proposed that EGFL6 mediates a paracrine mechanism of cross-talk between osteoblastic-like cells and vascular endothelial cells to regulate angiogenesis in the local bone environment. Osteoblast-like cells express EGFL6, which promotes endothelial cell migration by activating extracellular regulated protein kinases (ERK) 3,26. Studies have shown that the RGD peptides in the EGFL6 protein can affect EGFL6-induced endothelial cell migration. EGFL6 may also interact with integrin and regulate angiogenic activity 26.

### EGFL6 up-regulates the expression and proliferation of adipose tissue-derived stromal vascular cells in human obesity

Adipose-derived stromal vascular fraction (SVF) cells are a heterogeneous cell population with characteristics of stem cells. SVF cells have strong potential for regeneration, supporting processes such as angiogenesis, tissue remodeling, and immune regulation. Oberauer and colleagues 27 found that EGFL6 is up-regulated in human obesity and promotes proliferation of adipose tissue-derived stromal vascular cells. The expression of EGFL6 in subcutaneous adipose tissue increases significantly with obesity and decreases after weight loss. With the differentiation of human adipocytes *in vitro*, the expression and secretion of EGFL6 increases. This result indicates that mature adipocytes are the main source of EGFL6. Studies show that EGF-like repeats of EGFL6 mediate the specific adhesion to the surface of SVF cells in an RGD-dependent manner. These results characterizing a relationship between EGFL6 and SVF cells suggest that EGFL6 may be involved in angiogenesis and tissue reconstruction.

**Figure 2:**
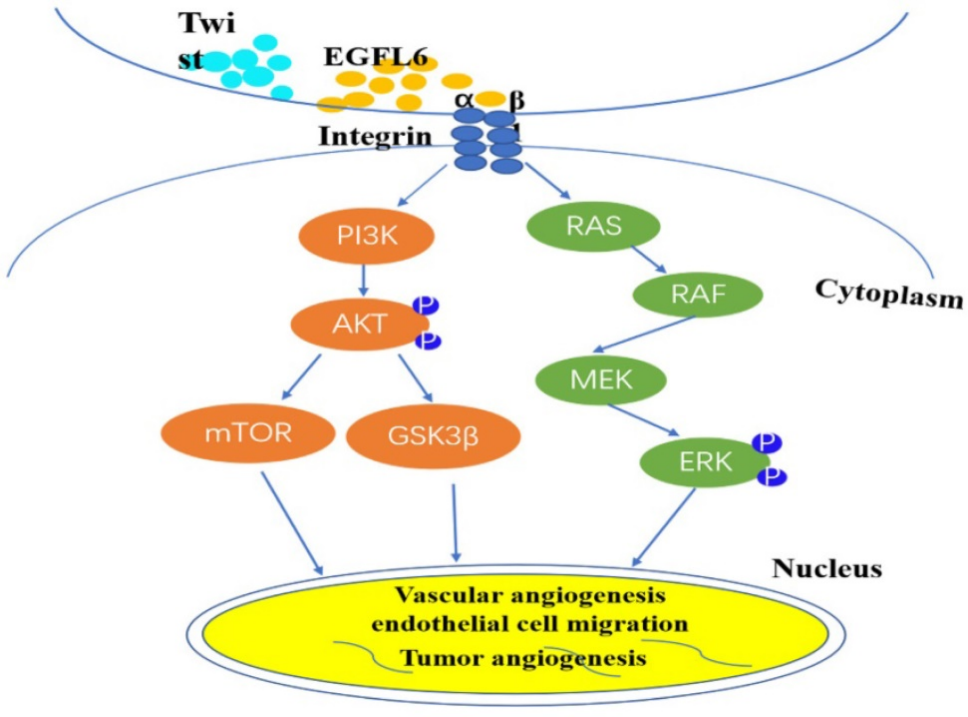
EGFL6 regulates angiogenesis by activating Akt/ERK signaling. Integrin β1 combines with RGD domain of EGFL6 to activate Akt/ERK signal pathway. Concurrently, Twist1 transcription factor can be combined with EGFL6 promoter to increase the transcription activity of EGFL6 and further enhance hypoxia-triggered angiogenesis. EGFL6 plays a role in angiogenesis, endothelial cell migration and tumor angiogenesis through Akt/ERK signaling pathway.

Based on this identification of the unique role of EGFL6 in tumor angiogenesis, more researchers are focusing on the anti-tumor effect of inhibiting tumor angiogenesis by blocking EGFL6. A major difference between tumors and wounds is the extent of hypoxia 28-30. Noh and colleagues sought to investigate the effects of differential expression of EGFL6 in tumor and wound endothelial cells 31. They created hind limb ischemia in mice by ligating the femoral artery in the hind limb of these mice 32. Results showed that the level of EGFL6 in tumor-associated endothelial cells of ischemic tissue was significantly higher than that in corresponding tissue without ischemia. The activity of the EGFL6 promoter was significantly higher under hypoxic conditions than under conditions with a normal level of oxygen. Analysis of the EGFL6 promoter showed that the Twist1 transcription factor binds the EGFL6 promoter and the transcriptional activity level of EGFL6 increases with the ectopic expression of TWIST1. These results further support the conclusion that there is a functional connection between TWIST1 and EGFL6. TWIST1 enhances EGFL6 for hypoxia-triggered angiogenesis. The structure of EGFL6 contains an RGD motif, and the RGD motif in EGFL6 can interaction with integrins. Noh et al. respectively silenced Tie2 and integrin expression by small interfering RNA technology, confirmed that EGFL6 regulates Tie2/AKT signaling through α5β1 integrin to promote tumor-associated endothelial cell migration and tube formation 31. And understanded of EGFL6 function in triggering integrin/Tie2/AKT signaling, which is a potent angiogenesis regulating signaling axis. So, EGFL6 participates in tumor angiogenesis through mediated Tie2/PI3K/AKT signals. These elements form a signal axis for effective angiogenesis regulation (Figure 2).

### Effect of EGFL6 on tumorigenesis and development

**Figure 3:**
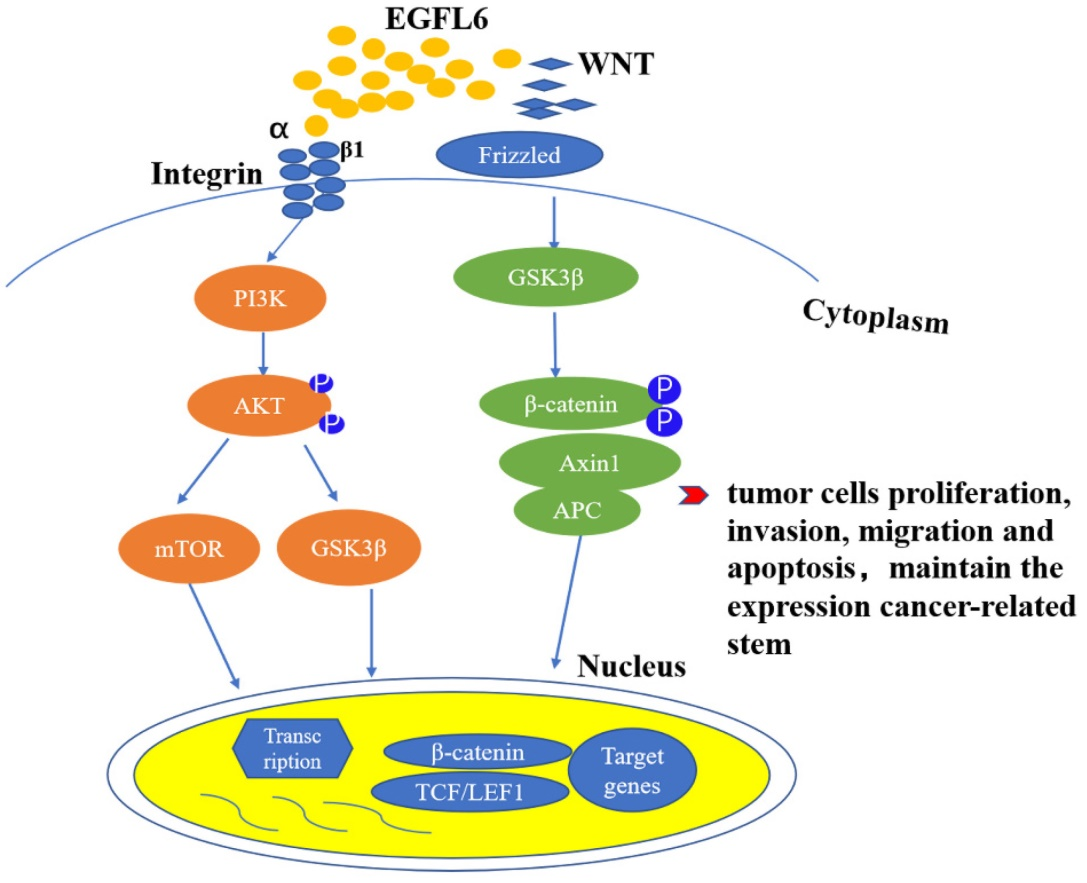
EGFL6 promotes tumor development by activating Wnt/β-catenin and Akt/ERK signaling pathways. Wnt/β- catenin and PI3K/Akt signaling pathways are closely related to the occurrence and development of tumors. RGD domain on EGFL6 activates PI3K/Akt signaling pathway by combination with integrin β1. EGFL6 can also activate Wnt/β- catenin pathway by reducing β-catenin and its downstream target TCF.

Invasion and metastasis of tumors involve a series of complicated processes, including cell adhesion, migration, invasion, angiogenesis, and adherent-independent growth 33-36 (Figure 3). The degradation of extracellular matrix (ECM) is also a key component for cancer cells to enter blood vessels and lymphatic vessels. The epidermal growth factor (EGF) superfamily has a series of conserved cysteines and glycines, which are located in the domain of 30 to 40 residues. They also have multiple EGF repeat sequences 4. Secretory cell surface molecules, they usually participate in the regulation of cell cycle, proliferation and development processes 37,38. EGFL6, as a member of the epidermal growth factor superfamily, is structurally similar to other members of the superfamily, and also has unique structural characteristics. It can regulate angiogenesis through multiple signal pathways. Studies have shown that EGFL6 is overexpressed in various tumor tissues, but not expressed or poorly expressed in normal tissues. Thus, the expression of EGFL6 in tumors suggests that it may also be related to the occurrence and development of cancer 29,30,39-43.

### EGFL6 and breast cancer

Since EGFL6 is highly expressed in tumor vessels but does not affect the normal wound healing, researchers have proposed EGFL6 as a new target for targeted inhibition of tumor vessels. Larimer and colleagues discovered ligands related to EGFL6 in tumor tissues of breast cancer through phage display 40. Their results suggest EGFL6 may play a role in angiogenesis of breast cancer. An and colleagues 43 reported *in vivo* and *in vitro* experiments whose results further confirmed that EGFL 6 can enhance the invasion and metastasis of breast cancer cells and stimulate tumor angiogenesis. Further, their results indicated that EGFL6 can also induce epithelial-mesenchymal transformation (EMT) of breast cancer and maintain the expression of breast cancer-related stem cells. The expression of EGFL6 in breast cancer is related to tumor node metastasis (TNM) stages of breast cancer. Studies also show that the higher the malignant degree of breast cancer, the higher the expression of EGFL6. Various studies show that EGFL6 plays an important role in the occurrence and development of breast cancer 43.

### EGFL6 and ovarian cancer

EGFL6 promotes the growth and metastasis of ovarian cancer by promoting the migration and asymmetric division of cancer stem cells (CSC) in ovarian cancer, Bai and colleagues 39 found that EGFL6 is expressed in both tumor cells and vascular cells. Using a tumor vascular model, they found expression of EGFL6 in vascular endothelium is similar to that of tumor cell EGFL6, which can promote the growth of transplanted tumor. In addition, the expression of EGFL6 in the vascular endothelium is related to the increase of metastasis of cancer cells and primary cancer cells. Anti-EGFL6 can completely eliminate ovarian cancer cells from diffusing into the blood of the ovary, suggesting that EGFL6 may play a key role in the ovarian microenvironment. EGFL6 neutralizing antibody inhibits the growth and metastasis of ovarian cancer cells. EGFL6 can promote the occurrence and development of ovarian tumors.

### EGFL6 and colorectal cancer

EGFL6 plays an important role in the occurrence and development of colorectal cancer. High expression of EGFL6 is correlated with poor survival of colorectal patients 44. *In vitro* and *in vivo* experiments have shown that EGFL6 affects the proliferation of colorectal cancer cells, regulates cell cycle, and inhibits apoptosis. Currently, studies of EGFL6 in tumor focus on the role of EGFL6 acting through the ERK signaling pathway, affecting the occurrence and development of tumor. Zhang and colleagues 44 first linked the function of EGFL6 to the Wnt/β-catenin pathway in studying the potential mechanism of EGFL6 in colorectal cancer. They found that the deletion of EGFL6 reduced β-catenin and its downstream target TCF7L2. This result suggests that EGFL 6 can also play a role in tumors by activating Wnt/β-catenin pathway.

In addition, EGFL6 is overexpressed in oral squamous cell carcinoma 45, nasopharyngeal carcinoma 46, lung cancer 47, benign meningioma 41 and other tissues. Chuang and colleagues 45 investigated the relationship between plasma EGFL6 level and clinicopathological features of patients with oral squamous cell carcinoma. Their results suggest that EGFL6 plays an important role in the development of oral squamous cell carcinoma. This finding is of great significance for the treatment of oral squamous cell carcinoma. The detection of EGFL6 protein can be used as a tumor marker to predict risk for oral squamous cell carcinoma in patients without this disease. Zhu and colleagues 46 reported that EGFL 6 promotes the migration of nasopharyngeal carcinoma cells by activating AKT pathway. Chang and colleagues 47 reported that high expression of EGFL6 can be used as an indicator of poor prognosis in lung adenocarcinoma, especially in younger patients. Wang and colleagues 41 proposed that PI3K/Akt activation and integrin-mediated signaling pathway participate in the pathogenesis of benign meningioma and anaplastic meningioma. Further, their report indicated that EGFL6 is overexpressed in benign meningioma tissues and serum. The study of EGFL6 in various tumors shows that EGFL6 plays an important role in the occurrence and development of cancer.

### EGFL6 as a candidate target for inhibiting tumor angiogenesis

Tumor growth and metastasis require angiogenesis. Tumor growth depends on neovascularization to ensure a continuous supply of oxygen and nutrition. Therefore, tumors can be treated by inhibiting tumor angiogenesis. Noh and colleagues 31 isolated endothelial cells from ten high-grade serous ovarian cancers (HGSC), five normal ovarian tissues, and seven healing wound patient samples. RNA was isolated and subjected to genomic analyses. Ribonucleic acid separation and genome analysis were conducted. Results showed that EGFL6 was predominantly expressed in tumor endothelial cells, but not in normal ovary or wound endothelial cells. EGFL6 is highly expressed in ovarian cancer tissues, and participates in the development of ovarian cancer by stimulating ovarian tumor angiogenesis 39,40. Noh and colleagues 31 silenced the expression of EGFL6 in a wound healing mouse model. Analyses of effects showed that EGFL6 lead to a decrease in the KOV3ip1 ovarian tumor load and significant decreases in tumor tissue proliferation index and tumor microvascular density. In contrast with antagonism of tumor angiogenesis, blocking EGFL6 expression in wound tissue does not affect normal wound healing.

### The prospect of EGFL6 in future clinical application

EGFL6 has the potential to promote tumor growth and metastasis. The use of EGFL6 monoclonal antibody in ovarian cancer tumor model significantly reduces tumor angiogenesis 39. When EGFL6 neutralizing antibody is used in breast cancer tumor models *in vivo* and *in vitro*, the proliferation and migration of cancer cells reduced, and the growth of tumor is inhibited 43. Therefore, it is hypothesized that EGFL6 may be a promising therapeutic target in tumor diagnosis and treatment. Further exploration of the mechanism of action in human tumors and development of corresponding antibody drugs will provide new tumor diagnostic markers, prognostic markers, or therapeutic targets.

EGFL6, as a secreted protein, is highly expressed by tumor-associated endothelial cells and acted on endothelial cells during physiological and pathological angiogenesis to control the development of blood vessels. Its role in vascular development supports tumor angiogenesis in part by mediating ERK/AKT signals. EGFL6 is overexpressed in human tumor cells, has a regulatory role in some oncogenic signaling pathways, and is related to tumor growth, metastasis, and progression. Therefore, In-depth study of the mechanism of EGFL6 gene in the development of tumors may provide new ideas for the scientific research of cancer, at the same time find new targets for tumor genetic diagnosis, and provide a new scientific basis for the selection of effective drug targets and theoretical support; further cooperation with biopharmaceutical companies to develop specific antibodies against the EGFL6 gene, targeting tumor blood vessels without damaging normal blood vessels, for targeted therapy of cancer, and thereby reducing the metastasis rate and mortality of cancer patients To improve the survival of cancer patients.

## Abbreviations

EGFL6: Epidermal growth factor-like domain-containing protein 6
VEGF: Vascular endothelial growth factor
EGFL: Epidermal growth factor-like domain
ERK: extracellular signal-regulated kinase
NFĸB: nuclear factor kappa B
MAPK: mitogen-activated protein kinase
PKB: protein kinase B
RGD: arg-gly-asp integrin binding motif
SVF: stromal vascular fraction
CSC: cancer stem cells
